# The AGCES Classification System for Endometriosis: Integrating Adenomyosis with Genital and Extragenital Staging—An Expert Consensus Framework from the American & Global College of Endometriosis Specialists (AGCES)

**DOI:** 10.3390/jcm15082871

**Published:** 2026-04-10

**Authors:** Camran Nezhat, Zahra Najmi, Vahid Monfared, Azadeh Nezhat, Ceana Nezhat, Farr Nezhat

**Affiliations:** 1 Camran Nezhat Institute, Center for Minimally Invasive and Robotic Surgery, Woodside, CA 94061, USA; 2 Stanford University Medical Center, Palo Alto, CA 94305, USA; 3 University of California, San Francisco, CA 94143, USA; 4 Department of Computer Science, Boston University, Commonwealth Ave., Boston, MA 02215, USA; 5 Nezhat Medical Center, Atlanta Center for Special Minimally Invasive Surgery and Reproductive Medicine, Atlanta, GA 30342, USA; 6 Nezhat Surgery for Gynecology/Oncology, Manhattan, NY 10128, USA; 7 Department of OB/GYN, NYU Long Island School of Medicine, Mineola, NY 11501, USA; 8 Department of OB/GYN, Weil Cornell Medical College of Cornell University, New York, NY 10065, USA

**Keywords:** endometriosis, adenomyosis, classification, staging, deep infiltrating endometriosis, surgical planning, web-based applications, AGCES (American & Global College of Endometriosis Specialists)

## Abstract

**Background:** Current endometriosis classification systems have important limitations in accurately describing total disease burden and predicting clinical outcomes. Existing staging frameworks often fail to integrate adenomyosis and do not adequately distinguish between genital and extragenital disease involvement. The aim of this article was to introduce the AGCES (American & Global College of Endometriosis Specialists) classification system, a novel framework designed to provide a more comprehensive and clinically meaningful approach to staging endometriosis. **Methods:** The AGCES classification system was developed through an expert consensus process involving scientific members of the American & Global College of Endometriosis Specialists (AGCES), informed by extensive surgical experience on thousands of endometriosis surgeries, synthesis of published evidence on disease pathophysiology and anatomical distribution, and systematic analysis of the limitations of existing classification systems (rASRM, ENZIAN, AAGL, EFI). **Results:** The framework integrates adenomyosis as a component of endometriosis staging and separates genital and extragenital disease into independent staging categories. Disease burden is reported using three parallel components representing adenomyosis (A), genital endometriosis (G), and extragenital endometriosis (E). A standardized operative reporting template and digital implementation through web-based applications were also developed to support clinical use. **Conclusions:** The AGCES classification system introduces a novel approach to endometriosis staging by integrating adenomyosis and separating genital and extragenital disease components. This framework provides a more complete assessment of disease burden and has the potential to improve clinical documentation, surgical planning, and research standardization in endometriosis care.

## 1. Introduction

Endometriosis affects at least 10% of reproductive-aged women and remains one of the most challenging conditions in gynecologic practice [1]. Despite its prevalence and significant impact on quality of life, fertility, and healthcare costs, the classification of endometriosis has remained problematic [2]. Current classification systems are based primarily on surgical observations and fertility outcomes, yet none adequately capture the full spectrum of disease presentation or reliably correlate with symptom severity or treatment outcomes [3,4].

The revised American Society for Reproductive Medicine (rASRM) classification, introduced in 1985 and revised in 1996, stratifies disease from Stage I (minimal) to Stage IV (severe) based on numerical scores for extent of disease and adhesions [5,6]. While widely adopted, this system has well-documented limitations: it does not predict treatment outcomes, fails to correlate with pain severity, and inadequately addresses deep infiltrating endometriosis [7,8]. The ENZIAN classification was subsequently developed to better characterize deep infiltrating disease affecting retroperitoneal structures, but it too lacks correlation with symptom severity [9]. The American Association of Gynecological Laparoscopists (AAGL) classification attempts to score intraoperative surgical complexity, while the Endometriosis Fertility Index (EFI) represents the only system specifically designed to predict non-IVF fertility outcomes following surgical intervention [10,11].

The World Endometriosis Society 2017 consensus statement recommended using a ‘classification toolbox’ combining the rASRM and ENZIAN systems to improve disease characterization [12]. The recent revision of the ENZIAN classification (#ENZIAN) has further refined the mapping of deep infiltrating endometriosis by incorporating peritoneal and ovarian disease compartments, representing an important step toward more comprehensive staging [9]. However, the lack of a universally adopted classification system that comprehensively captures disease burden while providing clinically relevant prognostic information represents a significant gap in endometriosis management.

In this study, we present the development and rationale for the AGCES classification system, first published in NPJ Women’s Health (Nature portfolio) in 2025 [13], a novel staging framework that addresses the fundamental limitations of existing classifications. We describe the system’s structure, staging criteria, implementation tools, and discuss its potential applications in clinical practice, surgical planning, and research standardization. Prospective multicenter validation is planned as the next phase of investigation.

## 2. The AGCES Classification System: Structure and Methodology

### 2.1. Development Approach

The AGCES classification system was developed to be applied based on preoperative imaging, intraoperative observations, and post operative histology confirmation, through an iterative consensus process conducted among scientific members of the American & Global College of Endometriosis Specialists (AGCES). The development process incorporated: (1) synthesis of the collective surgical experience of AGCES members across thousands of endometriosis cases spanning all stages and anatomic compartments, including complex cases involving deep infiltrating disease, multiorgan involvement, and concurrent adenomyosis, to verify that the system meaningfully distinguished patients with different disease burdens, surgical requirements, and clinical trajectories; (2) systematic review of the existing classification systems (rASRM, ENZIAN, AAGL, EFI) to identify specific limitations in disease characterization, including the exclusion of adenomyosis, the conflation of genital and extragenital disease, and poor correlation with surgical complexity; and (3) integration of published evidence from imaging, pathological, and molecular literature to anchor staging thresholds to established diagnostic parameters.

This article reports the development and clinical rationale of the AGCES framework. Formal validation through prospective multicenter application, inter-observer reliability testing, and correlation with clinical outcomes is planned as the next phase of investigation.

### 2.2. System Overview

The AGCES classification system categorizes endometriosis into three independent components; each staged from I to IV based on disease severity and location (Stage I (minimal), Stage II (mild), Stage III (moderate), Stage IV (severe)) (Table 1):

A (Adenomyosis): Stage I–IV, G (Genital Endometriosis): Affecting uterine serosa, ovaries, fallopian tubes, cervix, vulva, and vagina—Stage I–IV, and E (Extragenital Endometriosis): Affecting bladder, inguinal canal, pelvic sidewall, ureter, nerve, rectovaginal space, bowel and appendix, diaphragm, lung and/or other non-reproductive organs—Stage I–IV.

### 2.3. Overall AGCES Classification: Report as A__ G__ E__ (e.g., A3 G2 E4)

**Figure 1:**
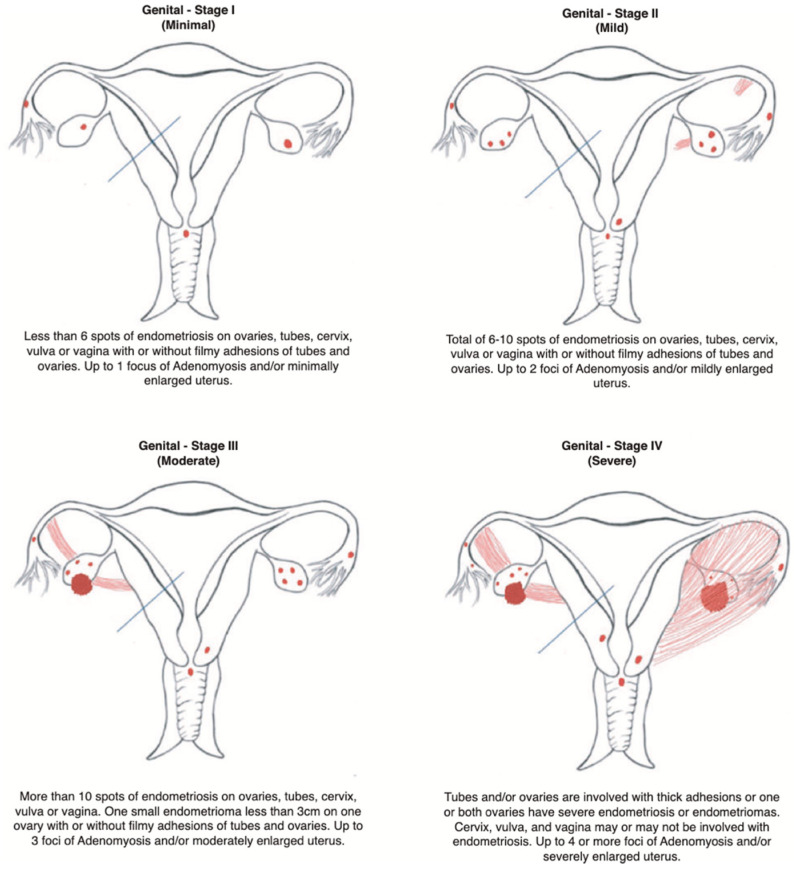
AGCES Classification System—Staging Criteria for Genital Endometriosis.

**Figure 2:**
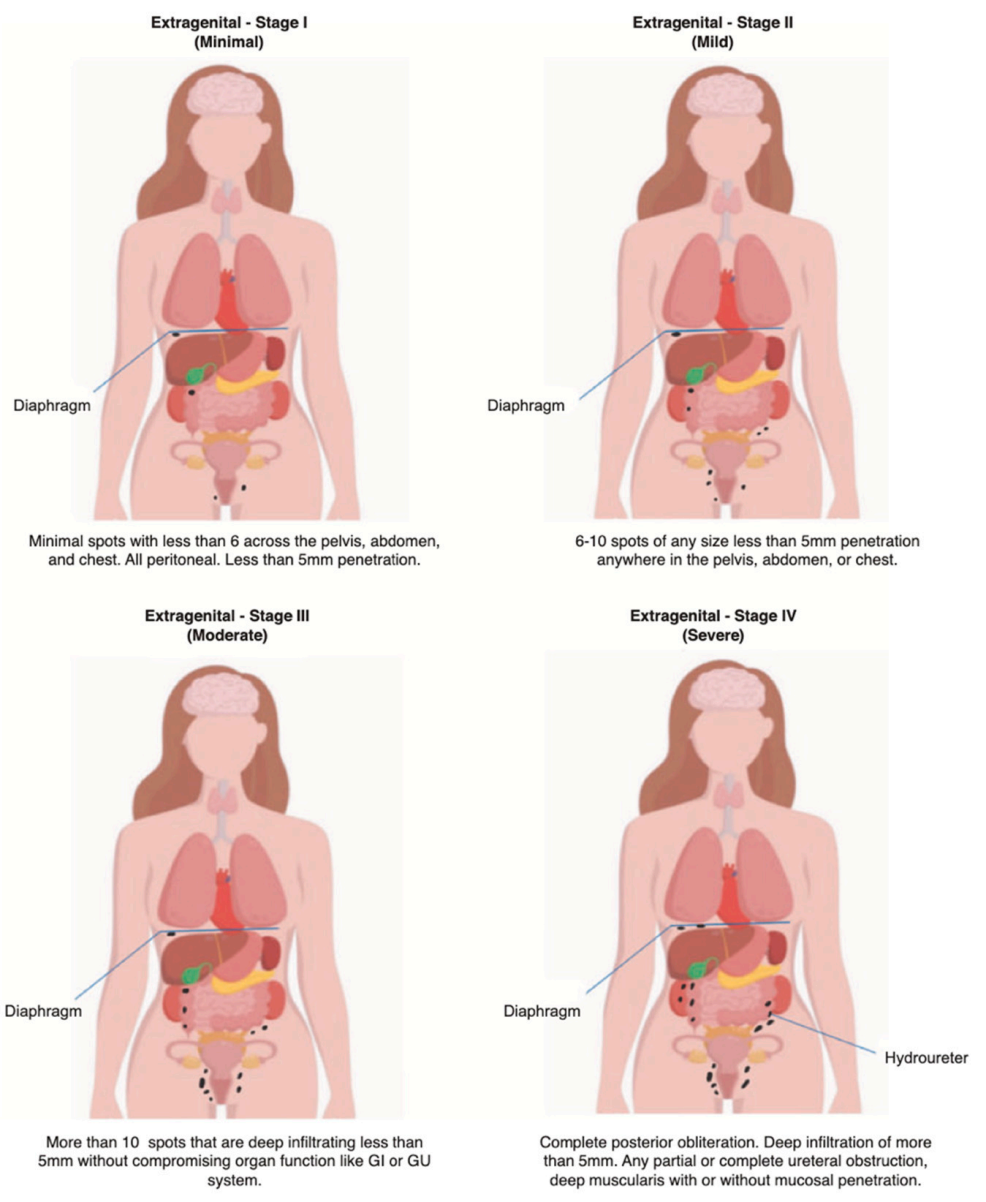
AGCES Classification System—Staging Criteria for Extra genital Endometriosis.

Disease burden is reported using a three-component designation, for example: ‘A2 G3 E2’ (Adenomyosis Stage 2, Genital endometriosis Stage 3, Extragenital endometriosisStage 2). This tripartite reporting provides an immediately comprehensible summary of total disease burden while maintaining granular information about disease distribution across all anatomical compartments (Figure 1 and Figure 2).

#### 2.3.1. Adenomyosis Staging Criteria (A)

Adenomyosis staging is based on any of these three categories: diffuse uterine wall thickening, diffuse junctional zone (JZ) thickening, and focal adenomyosis/adenomyomas, based on preoperative imaging measurements [14]. The highest stage across all three categories determines the overall adenomyosis classification. It should be acknowledged that imaging criteria for adenomyosis can vary between centers depending on operator experience, equipment, and imaging modality (ultrasound versus MRI), and standardization of imaging protocols would strengthen the reliability of this staging component. Although imaging modalities are valuable for the staging of adenomyosis, definitive diagnosis remains based on histopathological examination. This is typically established in cases of hysterectomy or when uterine biopsy specimens are obtained during surgery. Within the AGCES classification system, adenomyosis is incorporated as an independent parameter and contributes to staging only when present; its inclusion does not imply a prerequisite or progressive relationship with endometriosis.

Diffuse Uterine Wall Thickening:

Stage I: One wall affected, ≤20 mm thick

Stage II: Two walls ≤ 20 mm OR one wall > 20–30 mm

Stage III: One wall > 30 mm OR two walls > 20–30 mm

Stage IV: Two walls > 30 mm OR all uterus globally enlarged

Diffuse Junctional Zone Thickening: (JZ max is defined as the maximum measured thickness of the junctional zone.)

Stage I: JZ max > 6–8 mm and Diffuse JZ < 50% uterus

Stage II: JZ max > 8 mm and Diffuse JZ < 50% uterus

Stage III: Diffuse JZ > 50–80% of uterus

Stage IV: Diffuse JZ ≥ 80% of uterus

These thresholds follow a graduated severity model consistent with how asymmetric myometrial thickening is reported in the adenomyosis imaging literature, reflecting progressive uterine disruption that correlates with increasing surgical complexity and symptom burden [14].

Focal Adenomyosis/Adenomyoma:

Stage I: One lesion ≤ 10 mm

Stage II: ≥2 lesions ≤ 10 mm OR one lesion > 10–20 mm

Stage III: ≥2 lesions > 10–20 mm OR one lesion > 20 mm

Stage IV: ≥2 lesions > 20 mm

The 10 mm and 20 mm lesion size thresholds represent clinically meaningful increments in focal adenomyosis burden, reflecting increasing disruption of the myometrial architecture and progressively greater surgical complexity for uterine-sparing procedures.

#### 2.3.2. Genital Endometriosis Staging Criteria (G)

Genital endometriosis encompasses a disease affecting the uterine serosa, ovaries, fallopian tubes, cervix, vulva, and vagina. Staging is based on the number of spots, adhesion type, and presence/size of endometriomas, as assessed by the surgeon during laparoscopy. Spots refer to individually identifiable endometriotic implants; clustered lesions within a single anatomical site should be counted as individual spots where they are visually distinguishable:

Stage I: ≤6 endometriotic spots, absent or filmy adhesions, no endometriomas

Stage II: >6–10 spots, absent or filmy adhesions, no endometriomas

Stage III: >10 spots OR one endometrioma ≤ 30 mm (single ovary), absent or filmy adhesions

Stage IV: Bilateral endometrioma(s) OR endometrioma(s) > 30 mm OR thick/dense adhesions

Note: Stage IV criteria supersede spot count when present. The 3 cm endometrioma threshold reflects the widely used clinical decision-making cut-point at which surgical cystectomy is typically considered over conservative management. The filmy versus dense adhesion distinction follows established AFS/ASRM definitions [5,6,15].

#### 2.3.3. Extragenital Endometriosis Staging Criteria (E)

Extragenital disease affects non-reproductive structures including bladder, inguinal canal, pelvic sidewall, ureter, nerve, rectovaginal space, bowel and appendix, diaphragm, lung and/or other non-reproductive organs. Staging considers the number of spots, depth of penetration, cul-de-sac obliteration, and organ dysfunction, as assessed by the surgeon during laparoscopy:

Stage I: ≤6 spots, ≤5 mm depth (peritoneal), no cul-de-sac obliteration, no organ dysfunction

Stage II: 6–10 spots, ≤5 mm depth (peritoneal), no cul-de-sac obliteration, no organ dysfunction

Stage III: >10 spots, ≤5 mm depth (peritoneal), no cul-de-sac obliteration, no organ dysfunction

Stage IV: >5 mm depth OR muscularis OR mucosal invasion OR partial or complete cul-de-sac obliteration OR frozen pelvis OR organ dysfunction (ureteral/bowel obstruction OR other organ compromise)

Note: Stage IV criteria supersede spot count when present.

#### 2.3.4. Adhesion Classification

Pelvic adhesions are categorized as filmy (thin) or dense (thick) based on their structural and vascular characteristics, as established by the American Fertility Society and subsequently refined by the American Society for Reproductive Medicine [5,6]. Filmy adhesions are defined as thin, translucent, avascular or minimally vascular membranous bands that connect pelvic organs or peritoneal surfaces and can be easily separated by blunt dissection or gentle traction with minimal bleeding [5,6]. These adhesions are predominantly fibrinous in composition with limited collagen deposition and vascular ingrowth [15]. In contrast, dense adhesions are thick, opaque, highly vascularized fibrous bands that create firm attachments between structures and require sharp dissection for safe separation, often resulting in moderate to significant bleeding [15,16]. Dense adhesions contain substantial collagenous matrix with established vasculature and represent more organized, chronic scar tissue [15].

#### 2.3.5. Depth of Penetration

Deep infiltrating endometriosis (DIE) is defined as endometriotic lesions penetrating more than 5 mm beneath the peritoneal surface, a threshold established by Koninckx et al. [17] and widely accepted in the literature. Superficial endometriosis encompasses lesions limited to 5 mm or less of penetration. This distinction carries significant clinical implications: DIE exhibits distinct biological behavior compared to superficial implants [18], frequently involves retroperitoneal structures such as the bowel, bladder, and ureters, and typically requires advanced surgical expertise and multidisciplinary team involvement [18,19]. The AGCES system uses this established 5 mm threshold as the primary criterion distinguishing Stage I–III (superficial) from Stage IV (deep infiltrating) extragenital disease.

#### 2.3.6. Cul-de-Sac Obliteration

Obliteration of the posterior cul-de-sac represents one of the most challenging manifestations of advanced endometriosis, significantly impacting surgical complexity and patient outcomes. The pouch of Douglas, located between the posterior uterus and anterior rectum, becomes progressively compromised through adhesion formation, fibrotic changes, and infiltrative endometriotic tissue in severe disease presentations.

Partial obliteration is characterized by incomplete closure of the pouch of Douglas, where adhesions involve only part of the cul-de-sac depth, while leaving some patent peritoneal space remaining. In this presentation, the cul-de-sac is not completely filled or closed by adhesive disease, and residual peritoneal space remains accessible on examination. Band-like adhesions may traverse the space, creating partial compartmentalization while maintaining some degree of mobility between the rectum and posterior uterine structures. On transvaginal ultrasound, partial obliteration typically manifests as reduced but not completely absent “sliding sign,” indicating restricted rather than fixed anatomical relationships [20].

Complete obliteration represents the most severe form, defined as total closure and filling of the pouch of Douglas by dense adhesions, extensive fibrosis, or infiltrative endometriotic tissue, completely eliminating the normal peritoneal space between the posterior uterus/cervix and anterior rectum [21]. In complete obliteration, no patent peritoneal space remains, and dense adhesions create a fixed, immobile relationship between the rectal serosa and the posterior aspect of the uterus and vagina. This presentation is frequently associated with deep infiltrating endometriosis involving the rectovaginal space and may include nodular disease, extensive fibrosis, or both [18,22]. The complete loss of the “sliding sign” serves as a reliable diagnostic indicator of this severe anatomical distortion [20]. Surgical management of complete cul-de-sac obliteration requires meticulous dissection techniques and often necessitates multidisciplinary expertise, as the dense adhesive disease frequently involves multiple pelvic structures and obscures normal anatomical planes [23].

Frozen pelvis is a severe, advanced stage of deep infiltrating endometriosis (DIE) where extensive fibrotic adhesions cause pelvic organs—uterus, ovaries, fallopian tubes, bowel, and bladder—to become densely tethered together [9,17]. This “frozen” state, often characterized by obliteration of the pouch of Douglas [20,22], results from chronic inflammation and progressive fibrotic remodeling of the peritoneal surfaces [15]. The condition causes excruciating pain, infertility, and significant organ dysfunction, and its surgical management requires meticulous dissection of multiple involved compartments [9].

#### 2.3.7. Evidence Basis for Staging Thresholds

Each staging threshold in the AGCES system was selected based on published evidence rather than arbitrary numerical divisions. The 5 mm depth-of-penetration threshold distinguishing superficial from deep infiltrating endometriosis was established by Koninckx et al. [17] and is universally accepted in the endometriosis literature. Adenomyosis JZ thresholds (>6 mm, >8 mm) correspond to established imaging diagnostic criteria [14]. The endometrioma size threshold of 3 cm reflects the widely used clinical decision-making cut-point for surgical versus conservative management. Adhesion classification follows AFS/ASRM definitions [5,6,15]. Cul-de-sac obliteration and organ dysfunction criteria reflect recognized markers of advanced disease requiring multidisciplinary surgical management [18,20,21,22].

## 3. Novel Features of the AGCES Classification System

### 3.1. Integration of Adenomyosis

A fundamental innovation of the AGCES system is the integration of adenomyosis into endometriosis staging based on emerging evidence suggesting that the two conditions may share a common pathobiological basis. Recent molecular and histopathological studies have demonstrated that adenomyosis and endometriosis harbor similar somatic epithelial mutations and exhibit overlapping cellular composition [24,25], supporting the hypothesis that they may represent related manifestations of a single underlying disease process rather than entirely distinct entities. However, each condition can also occur independently. While this concept remains an area of active investigation and debate, the anatomical distinction between the two conditions—endometriosis involving ectopic endometrial tissue outside the uterine myometrium, and adenomyosis representing a similar pathological process within the myometrium—and their frequent clinical co-occurrence [26] provide a strong rationale for incorporating both into a unified staging framework. This integration is intended not as a definitive statement of shared etiology, but as a clinically pragmatic approach that captures total disease burden more completely than systems that exclude adenomyosis entirely.

Excluding adenomyosis from endometriosis staging systems, as all current classifications do, results in incomplete disease characterization and underestimation of total disease burden. Patients frequently present with both conditions simultaneously, yet existing systems fail to capture this concurrent presentation [26]. The separation of adenomyosis and endometriosis in classification schemes does not reflect the underlying pathophysiology and may compromise clinical decision-making, particularly regarding surgical planning and patient counseling.

The AGCES system addresses this limitation by incorporating adenomyosis as an integral component of disease staging. This integration acknowledges the biological continuum between myometrial and extrauterine endometriotic lesions while maintaining practical distinctions that inform surgical approach. For example, a patient presenting with minimal peritoneal disease but severe adenomyosis (A4 G1 E1) represents a fundamentally different clinical scenario from one with severe extragenital disease and minimal adenomyosis (A1 G1 E4), yet traditional classification systems would fail to capture this critical distinction.

### 3.2. Separation of Genital and Extragenital Components

The second major innovation involves separating genital and extragenital endometriosis into independent staging categories. This distinction reflects the clinical reality that these disease presentations require fundamentally different surgical approaches and expertise. Genital endometriosis primarily affects reproductive structures and is typically managed by gynecologic surgeons with standard laparoscopic techniques. In contrast, extragenital disease, particularly deep infiltrating lesions affecting the bowel, bladder, or ureters, often requires multidisciplinary surgical teams and advanced techniques including bowel resection, partial cystectomy, or ureterolysis [27,28,29,30].

Current classification systems conflate these distinct disease presentations, potentially obscuring important prognostic information. A patient with Stage IV disease by rASRM criteria might have bilateral ovarian endometriomas with dense adhesions (primarily genital disease) or severe rectovaginal space disease with bowel involvement (primarily extragenital disease). These presentations carry vastly different implications for surgical complexity, perioperative complications, and postoperative outcomes, yet traditional staging fails to distinguish between them [22,31].

By separately staging genital and extragenital components, the AGCES system is designed to provide critical information for surgical planning and informed consent. A classification of G4 E1 immediately signals that the surgical challenge primarily involves ovarian disease with adhesions, while G1 E4 indicates complex extragenital disease requiring multidisciplinary expertise. Although definitive staging is established during laparoscopy, preoperative imaging can suggest the likely extent of disease and guide preliminary operative strategy—for example, identifying bilateral endometriomas and suspected deep infiltrating disease allows anticipation of a complex case with prolonged operative time and possible multidisciplinary involvement. This granularity may facilitate appropriate resource allocation, surgical team selection, and realistic patient counseling regarding operative duration, complication risk, and recovery trajectory.

## 4. Clinical Applications and Advantages

### 4.1. Surgical Planning

The AGCES classification system is designed to enhance surgical planning by clearly describing disease distribution. Surgeons may better identify cases likely to require multidisciplinary teams (e.g., colorectal surgery for bowel involvement, urology for ureteral disease), anticipate operative complexity and duration, select the appropriate surgical setting and required equipment, and counsel patients regarding potential risks, recovery expectations, and the likelihood of additional procedures. For instance, a patient classified as A3 G2 E4 would likely benefit from multidisciplinary surgical team availability and extended operative time allocation. Conversely, A1 G2 E1 disease might be appropriate for a standard diagnostic laparoscopy with limited extragenital involvement requiring basic excision techniques [32,33].

### 4.2. Interdisciplinary Communication

The tripartite AGCES designation may facilitate clear communication among healthcare providers managing endometriosis patients. The concise notation (e.g., A2 G3 E2) immediately conveys disease distribution to radiologists interpreting imaging studies, pain management specialists developing treatment plans, fertility specialists counseling regarding reproductive options, and colorectal or urologic surgeons evaluating extragenital disease burden [34].

This standardized communication framework may reduce ambiguity inherent in descriptive reporting and help ensure all team members share a common understanding of disease severity across all anatomical compartments. The system’s structure may also facilitate research collaboration by enabling precise categorization of patient populations for comparative outcome studies.

### 4.3. Outcome Reporting and Quality Improvement

Standardized staging may enable systematic tracking of surgical outcomes stratified by disease burden. Institutions can analyze complication rates, operative duration, symptom improvement, and recurrence patterns across specific AGCES patterns. This granular outcome data could facilitate identification of best practices, benchmarking against peer institutions, and continuous quality improvement in endometriosis care delivery.

The separate staging of adenomyosis may prove particularly valuable for tracking uterine preservation versus hysterectomy rates stratified by adenomyosis severity, correlating adenomyosis stage with pain improvement following conservative surgery, and identifying optimal management strategies for combined adenomyosis-endometriosis presentations.

### 4.4. Relationship to Existing Classification Systems

The AGCES system is not proposed as a replacement for the rASRM or ENZIAN classifications, but rather as a complementary framework designed to function within the “classification toolbox” approach recommended by the World Endometriosis Society 2017 consensus statement [12]. Each system contributes distinct clinical information: the rASRM classification provides a familiar overall severity score with decades of comparative research data, the ENZIAN classification offers detailed retroperitoneal compartment mapping for deep infiltrating disease, and the AGCES system adds adenomyosis staging and independent characterization of genital versus extragenital disease burden—dimensions that neither existing system captures. In practice, these systems may be reported in parallel; for example, a patient might be documented as rASRM Stage IV, ENZIAN P2O2A1, AGCES A3 G4 E4, with each notation contributing a complementary layer of information to guide surgical planning, interdisciplinary communication, and research categorization. Whether this combined reporting approach provides independent prognostic value beyond existing classifications used alone, and whether clinicians find it practically feasible in routine surgical documentation, are questions that require prospective evaluation.

## 5. Implementation Tools

### 5.1. Standardized Operative Reporting Sheet

A comprehensive operative reporting worksheet has been developed to facilitate intraoperative documentation of AGCES staging (Supplementary Material). This structured form guides surgeons through systematic assessment of all relevant anatomical sites, ensuring complete disease characterization while reducing documentation burden. The worksheet includes sections for:

Detailed mapping of genital endometriosis (ovarian, tubal, cervical, vaginal involvement)

Comprehensive extragenital assessment (bladder, bowel, ureters, rectovaginal space, pelvic sidewall, diaphragm)

Adenomyosis evaluation (uterine size, wall thickness, focal lesion mapping)

The structured format is intended to promote consistent application of staging criteria across different surgeons and institutions, with the goal of reducing inter-observer variability and improving data quality.

The AGCES operative reporting framework has been applied in clinical practice. Following the initial introduction of the AGCES system in NPJ Women’s Health (Nature portfolio) [13], the classification and its standardized reporting approach were used to characterize disease burden in two subsequent published studies [35,36]. These applications demonstrate the worksheet’s feasibility and practical utility in both clinical documentation and research settings. Formal pilot testing—including structured assessment of completion time, ease of use, documentation completeness, and inter-observer consistency across multiple surgeons and institutions—is planned as a next step and has been noted in the Future Directions section.

### 5.2. Digital Implementation

A web-based application has been developed to support AGCES classification adoption: https://all-ways-web-app.lovable.app (accessed on 2 April 2026).

This digital tool is designed to provide interactive staging calculators with real-time stage determination, automated documentation generation of standardized operative reports, and data export capabilities for integration with electronic health records and research databases.

## 6. Limitations and Future Directions

While the AGCES classification system represents an advance in comprehensive disease characterization, several limitations warrant consideration. First, the staging thresholds, while anchored to published diagnostic criteria, have not yet been prospectively validated for their ability to discriminate between clinically distinct patient groups. Second, correlations between AGCES staging and clinical outcomes—including symptom severity, surgical complexity, complication rates, fertility outcomes, and recurrence—require evaluation through prospective, multicenter studies. Third, the interobserver reliability of staging assignments has not yet been formally assessed.

Future research should focus on validating the system’s ability to predict surgical complexity, perioperative complications, symptom improvement, and fertility outcomes. Additionally, investigating whether specific AGCES patterns correlate with distinct symptom profiles or differential responses to medical therapy would further enhance the system’s clinical utility.

Furthermore, prospective head-to-head comparison studies are needed to determine whether AGCES staging provides independent prognostic information beyond what the rASRM and ENZIAN classifications already capture, and whether the combined use of multiple classification systems improves clinical decision-making or introduces unnecessary documentation burden.

## 7. Conclusions

Developed through expert consensus among members of AGCES, the AGCES classification system addresses fundamental limitations of existing endometriosis classification schemes through two key innovations: integration of adenomyosis as a component of endometriosis staging based on their shared pathobiology, and separation of genital and extragenital disease into independent staging categories. By reporting disease using three distinct components (A__ G__ E__), this system provides comprehensive characterization of total disease burden while maintaining clinically relevant distinctions that inform surgical planning, facilitate interdisciplinary communication, and enable precise outcome reporting.

Prospective multicenter validation of the AGCES system’s reliability, clinical utility, and predictive capacity represents the essential next step toward potential adoption in clinical practice and research.

## Figures and Tables

**Table 1:** AGCES classification system—staging criteria. Adenomyosis, Genital Endometriosis, Extragenital Endometriosis.

Anatomical Site/Feature	Stage I (Minimal)	Stage II (Mild)	Stage III/(Moderate)	Stage IV (Severe)
* Adenomyosis, based on preoperative imaging (uterine wall thickening, JZ thickening, Focal adenomyosis)				
Diffuse uterine wall thickening	One wall affected, ≤20 mm thick	Two walls ≤ 20 mm OR one wall > 20–30 mm	One wall > 30 mm OR two walls > 20–30 mm	Two walls > 30 mm OR all uterus globally enlarged
Diffuse JZ thickening	JZ max > 6–8 mm AND Diffuse JZ thickening < 50% uterus	JZ max > 8 mm AND Diffuse JZ thickening <50% uterus	Diffuse JZ thickening > 50–80% of uterus	Diffuse JZ thickening ≥ 80% of uterus
Focal adenomyosis/Adenomyoma	One lesion ≤ 10 mm	≥2 lesions ≤ 10 mm OR One lesion > 10–20 mm	≥2 lesions > 10–20 mm OR One lesion > 20 mm	≥2 lesions > 20 mm
Genital Endometriosis, based on intraoperative findings (uterine serosa, ovaries, tubes, cervix, vulva, vagina)				
Number of endometriosis spots	≤6 spots	>6–10 spots	>10 spots	Variable **
Adhesions	None or filmy	None or filmy	None or filmy	Thick/dense
Endometrioma	Absent	Absent	One endometrioma ≤ 30 mm (single ovary)	Bilateral endometrioma(s) OR endometrioma(s) > 30 mm
Extragenital Endometriosis, based on intraoperative findings (Affecting bladder, inguinal canal, pelvic sidewall, ureter, nerve, rectovaginal space, bowel and appendix, diaphragm, lung and/or other non-reproductive organs)				
Number of endometriosis spots	≤6 spots	>6–10 spots	>10 spots	Variable **
Depth of penetration	≤5 mm (peritoneal)	≤5 mm (peritoneal)	≤5 mm (peritoneal)	>5 mm OR muscularis OR mucosal invasion
Cul-de-sac obliteration	None	None	None	Partial or complete
Frozen Pelvis	None	None	None	Anterior OR Posterior
Organ dysfunction	None	None	None	Ureteral/bowel obstruction OR other organ compromise

JZ max = Maximum junctional zone thickness. * For adenomyosis Staging: Use the highest score across all three adenomyosis categories (diffuse myometrium, diffuse JZ, focal, adenomyoma). ** Stage IV criteria supersede spot count when present (dense adhesions, bilateral or large endometriomas make it Stage IV of genital endometriosis and deep penetration, Cul-de-sac obliteration, Frozen Pelvis, and any Organ dysfunction make it Stage IV of extra genital endometriosis regardless of spot count).

## Data Availability

No new data were created or analyzed in this study.

## Supplementary Materials

The following supporting information can be downloaded at: https://www.mdpi.com/article/10.3390/jcm15082871/s1, ENDOMETRIOSIS-ADENOMYOSIS STAGING WORKSHEET.

## Abbreviations

The following abbreviations are used in this manuscript:

AGCES	American & Global College of Endometriosis Specialists
A	Adenomyosis
G	Genital Endometriosis
E	Extragenital Endometriosis
rASRM	Revised American Society for Reproductive Medicine
ENZIAN	ENZIAN classification system (deep infiltrating endometriosis classification)
AAGL	American Association of Gynecological Laparoscopists
EFI	Endometriosis Fertility Index
IVF	In Vitro Fertilization
JZ	Junctional Zone
JZ max	Maximum Junctional Zone thickness
DIE	Deep Infiltrating Endometriosis
