# Supplementary material for: The AGCES Classification System for Endometriosis: Integrating Adenomyosis with Genital and Extragenital Staging—An Expert Consensus Framework from the American & Global College of Endometriosis Specialists (AGCES)
Source: J Clin Med. 2026 Apr 10;15(8):2871. doi: 10.3390/jcm15082871 (PMC13116373; doi:10.3390/jcm15082871)
Supplement: Supplementary file 1 [file jcm-15-02871-s001.zip › jcm-4194708-supplementary.pdf]

ENDOMETRIOSIS-ADENOMYOSIS STAGING WORKSHEET
AGE Classification System

**Patient Name:**	____________________	**MRN:**	____________________
**Date of Surgery:**	____________________	**Surgeon:**	____________________

SECTION A: ADENOMYOSIS

A1. DIFFUSE UTERINE WALL THICKENING

**Anterior wall:**	☐ Yes ☐ No Thickness: _____ mm
**Posterior wall:**	☐ Yes ☐ No Thickness: _____ mm

Stage: ☐ 1 (One wall ≤20mm) ☐ 2 (Two walls ≤20mm OR one >20-30mm) ☐ 3 (One >30mm OR two >20-30mm) ☐ 4 (Two >30mm OR global)

A2. DIFFUSE JZ THICKENING

**JZ max thickness:**	_____ mm ☐ <50% uterus ☐ 50-80% uterus ☐ ≥80% total JZ

Stage: ☐ 1 (JZmax >6-8mm AND diffuse thickening <50%) ☐ 2 (JZmax >8mm AND diffuse thickening <50%) ☐ 3 (Diffuse thickening 50-80%) ☐ 4 (thickening ≥80% JZ infiltration)

A3. FOCAL ADENOMYOSIS (ADENOMYOMAS)

#	Location	Size (mm)	Description
1	☐ Ant ☐ Post ☐ Fund ☐ Lat	_____	
2	☐ Ant ☐ Post ☐ Fund ☐ Lat	_____	
3	☐ Ant ☐ Post ☐ Fund ☐ Lat	_____	
4	☐ Ant ☐ Post ☐ Fund ☐ Lat	_____	

TOTAL FOCI: _____ Stage: ☐ 1 (One ≤10mm) ☐ 2 (≥2 ≤10mm OR one >10-20mm) ☐ 3 (≥2 >10-20mm OR one >20mm) ☐ 4 (≥2 >20mm)

ADENOMYOSIS STAGE (A): _______ (Use HIGHER stage from A1, A2, A3)

SECTION G: GENITAL ENDOMETRIOSIS

(Ovaries, tubes, cervix, vulva, vagina)

G1. NUMBER OF ENDOMETRIOSIS SPOTS

Location	# Spots	Description
Right Ovary	_____	
Left Ovary	_____	
Right Tube	_____	
Left Tube	_____	
Cervix	_____	
Vulva	_____	
Vagina	_____	
**TOTAL:**	_____	☐ Stage I (≤6) ☐ Stage II (>6-10) ☐ Stage III (>10)

G2. ADHESIONS

Location	Type
Right Ovary	☐ None ☐ Filmy ☐ Dense
Left Ovary	☐ None ☐ Filmy ☐ Dense
Right Tube	☐ None ☐ Filmy ☐ Dense
Left Tube	☐ None ☐ Filmy ☐ Dense

Adhesion Type: ☐ None/Filmy (Stage I-III) ☐ Thick/Dense (Stage IV)

G3. ENDOMETRIOMAS

Location	Present?	Size (cm)	Number
Right Ovary	☐ Yes ☐ No	_____	_____
Left Ovary	☐ Yes ☐ No	_____	_____
**Classification: ☐ Absent (I-II) ☐ ≤3cm unilateral (III) ☐ >3cm or bilateral (IV)**			

GENITAL ENDOMETRIOSIS STAGE (G): _______

SECTION E: EXTRAGENITAL ENDOMETRIOSIS

(Bladder, bowel, ureter, RVS, pelvic sidewall, diaphragm, appendix)

E1. NUMBER OF EXTRAGENITAL SPOTS

Location	Number	Description
Bladder	_____	
Bowel (Rectum/Sigmoid)	_____	
Right Ureter	_____	
Left Ureter	_____	
Rectovaginal Septum	_____	
Pelvic Sidewall	_____	
Diaphragm	_____	
Appendix	_____	
Other: _____________	_____	
**TOTAL:**	_____	☐ Stage I (≤6) ☐ Stage II (>6-10) ☐ Stage III (>10)

E2. DEPTH OF PENETRATION

**Deepest: ☐ ≤5mm (I-III) ☐ >5mm OR muscularis/mucosal (IV)**

E3. CUL-DE-SAC OBLITERATION

**Visual/Palpation:**	☐ Patent ☐ Partially obliterated ☐ Completely obliterated
**Status: ☐ None (I-III) ☐ Partial/Complete obliteration (IV)**	

EXTRAGENITAL ENDOMETRIOSIS STAGE (E): _______

FINAL AGE CLASSIFICATION: A___ G___ E___ OVERALL STAGE (highest): _______
